# Design, Synthesis and Cytotoxic Activities of Novel Aliphatic Amino-Substituted Flavonoids

**DOI:** 10.3390/molecules181114070

**Published:** 2013-11-13

**Authors:** Guannan Liu, Zhen Ge, Mengdan Zhao, Yifeng Zhou

**Affiliations:** 1 College of Life Sciences, China Jiliang University, Hangzhou 310018, Zhejiang, China; 2 Hangzhou Hertz Pharmaceutical Co., Ltd., Hangzhou 310018, Zhejiang, China; E-Mail: gez@hzpharma.com; 3 Women’s Hospital, School of Medicine, Zhejiang University, Hangzhou 310006, Zhejiang, China; E-Mail: mengdanzhao@126.com

**Keywords:** flavonoid, chalcone, synthesis, cytotoxic activity

## Abstract

A series of flavonoids **9a**–**f**, **13b**, **13d**, **13e** and **14a**–**f** bearing diverse aliphatic amino moieties were designed, synthesized and evaluated for their cytotoxic activities against the ECA-109, A-549, HL-60, and PC-3 cancer cell lines. Most of the compounds exhibited moderate to good activities. The structure-activity relationships were studied, revealing that the chalcone skeleton is the most preferable for cytotoxic activities. Chalcone **9d** was the most promising compound due to its high potency against the examined cancer cell lines (its IC_50_ values against ECA-109, A549, HL-60 and PC-3 cells were 1.0, 1.5, 0.96 and 3.9 μM, respectively).

## 1. Introduction

**Figure 1:**
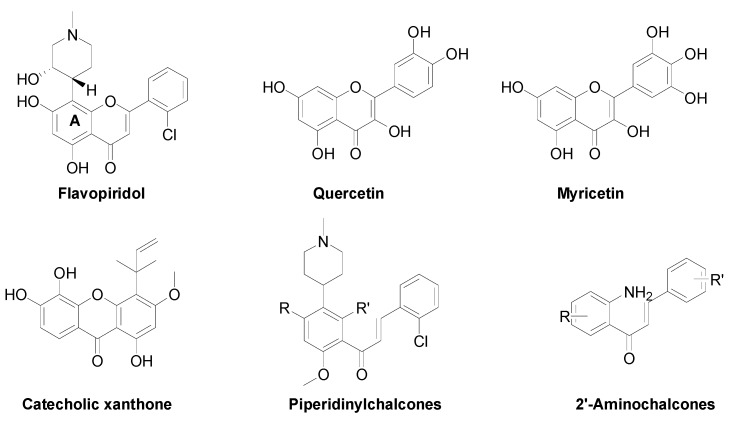
Structures of flavonoids with cytotoxic activity.

Flavonoids are a class of privileged structures that display a remarkable spectrum of biological activities [1]. Flavonoids are divided into six main categories: flavonols, flavones, flavanones, catechins, isoflavones and anthocyanidins, based on differences in their molecular backbone structures. A growing number of studies have indicated that flavonoids have important chemopreventive and chemotherapeutic effects on cancer [2,3]. Flavonoids, such as quercetin, catecholic xanthone and myricetin (Figure 1), show remarkable chemopreventive activities in a variety of bioassay systems and animal models [4,5,6]. More importantly, flavopiridol (Figure 1) that was identified as the first cyclin-dependent kinase (CDK) inhibitor has been tested in phase II clinical trials for the treatment of cancer [7,8].

The structure-activity relationship (SAR) studies of flavopiridol revealed that the presence of the aliphatic amino on A ring is very important for CDK inhibitory activity [9]. On the other hand, the structural modifications of flavonoids with the introduction of nitrogen atoms have attracted considerable attention due to the observed improvements of the biological activities [10,11,12,13]. For example, 2'-aminochalcones demonstrated a significantly increased antitumor activity compared with chalcones that lack this function [11]. Another investigation showed that the introduction of aliphatic amino groups onto the flavonoids scaffold resulted in potent antitumor compounds [13].

**Figure 2:**
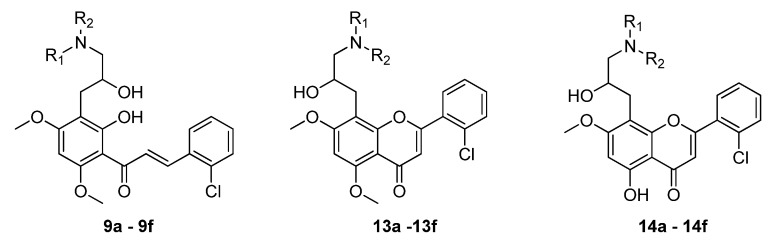
Structures of synthesized compounds **9**, **13** and **14**.

Moreover, the functional aliphatic amino groups can enhance the aqueous solubility and drug-like character of candidate compounds [12,13]. Based on these observations, it is of interest to further evaluate the roles of basic amino functions in the antitumor activity of flavonoids. We speculated that introducing diverse flexible aliphatic amino groups to the flavonoid scaffold might be an effective way in discovering novel flavonoids with potential bioactivities. In this paper, we describe the detailed synthetic routes to the novel aliphatic amino substituted flavonoids **9**, **13** and **14** (Figure 2) and their biological activities against ECA-109, A-549, HL-60, and PC-3 cancer cell lines. Moreover, we discuss the corresponding structure-activity relationships.

## 2. Results and Discussion

### 2.1. Chemistry

#### 2.1.1. Synthesis of Chalcone Derivatives **9a**–**f**

**Scheme 1:**
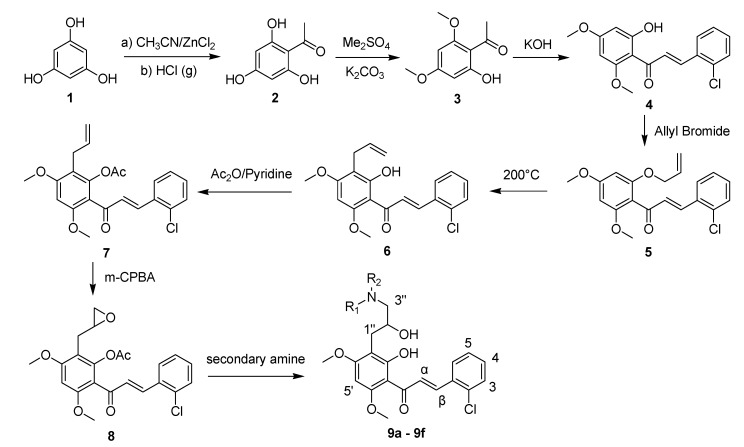
Synthesis of chalcone derivatives **9a**–**f**.

The preparation of the target chalcones **9a**–**f** is depicted in Scheme 1. Briefly, substrates **2** [14] and **3** [15] were prepared according to the described procedures. The dimethylated intermediate **3** was then treated with an equimolar amount *o*-chlorobenzaldehyde in a base-catalyzed Claisen-Schmidt reaction to afford chalcone **4** in good yield [16]. With the chalcone core established, all that remained was the introduction of the allyl group to position-3'. This involved *O*-allylation of chalcone **4** with allyl bromide, followed by Claisen rearrangement in *N*,*N*-dimethylaniline at 190 °C under N_2_. The position of the allyl function was confirmed by a nuclear Overhauser effect (NOE) experiment, which showed correlations between the OH-2' and H-1" signals. The synthesis of the target products **9a**–**f** was achieved in moderate yields by the triethylamine-catalyzed reaction of the epoxychalcone **8** with suitable secondary amines in acetonitrile at room temperature. It is worth noting that direct epoxidation of the allylchalcone **6** with *m*-chloroperoxybenzoic acid yielded no product. As expected, after protection of the phenolic hydroxyl with an acetyl group, the epoxychalcone was formed after treatment of intermediate **7** with *m*-chloroperoxybenzoic acid, and the pure product epoxychalcone **8** was obtained.

#### 2.1.2. Synthesis of Flavone Derivatives **13b**, **13d**, **13e** and **14a**–**f**

**Scheme 2:**
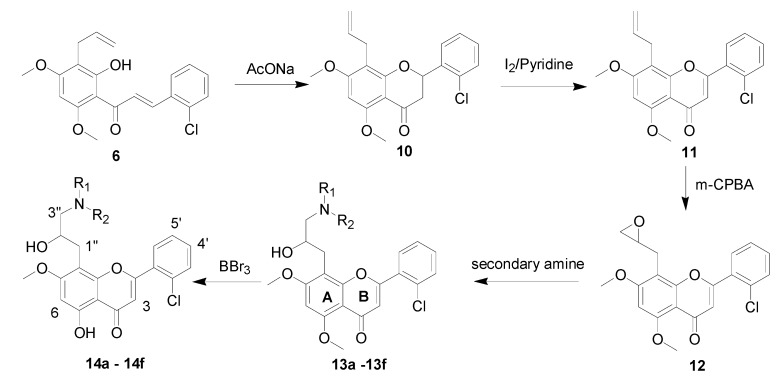
Synthesis of flavone derivatives **13b**, **13d**, **13e** and **14a**–**f**.

The general method for the synthesis of the desired flavone derivatives **13b**, **13d**, **13e** and **14a**–**f** is outlined in Scheme 2. The allylchalcone **6** was cyclized in the presence of sodium acetate in 95% ethanol to produce flavanone **10** [17]. Subsequent oxidation of flavanone **10** with iodine through a modification of the procedure previously reported for the synthesis of analogous compounds [18] generated flavone **11**. Preparation of epoxyflavone **12** was accomplished by the reaction of flavone **11** with *m*-chloroperoxybenzoic acid. Refluxing epoxyflavone **12** in ethanol with suitable secondary amines in the presence of triethylamine resulted in the formation of dimethoxyflavones **13a**–**f** in high yields. The final steps required for completion of the synthesis target flavones were the selective demethylation of the methyl ether-protecting groups. The cleavage of aryl methyl ethers to liberate the corresponding phenolic compounds has been reviewed previously in the literature [19,20,21]. Our experiments showed that deprotection of dimethoxylflavone using a 2-fold excess of boron tribromide at room temperature successfully provided monomethoxyflavones **14a**–**f** in good yields.

### 2.2. Biological Activities

The *in vitro* cytotoxic activities of the chalcone derivatives **9a**–**f** and flavone derivatives **13b**, **13d**, **13e** and **14a**–**f** against four cancer cell lines, namely human esophageal carcinoma cell (ECA-109), human lung adenocarcinoma cell (A-549), human leukemia cell (HL-60) and human prostate carcinoma cell (PC-3), were assayed by the MTT method using taxol as the positive control. Table 1 summarizes the results expressed as IC_50_ values.

For the derivatives bearing the same substituents, the chalcones derivatives **9a**–**f** had higher cytotoxic activity than the corresponding flavones **14a**–**f**, with all IC_50_ values lower than 10 µg/mL against the human tumor cell lines tested (Table 1). Compound **9b** with a piperidinyl substituent at position-3' displayed prominent cytotoxic activity, whereas introducing a morpholino or pyrrolidinyl substituent led to compounds **9a** and **9c**, respectively, which all demonstrated decreased cytotoxic activities. These results indicated that a smaller substituent and the introduction of additional heteroatoms were not suitable options for position-3'. Interestingly, further introducing open chain aliphatic amino groups afforded compounds **9d** (diethylamino), **9e** (ethylmethylamino) and **9f (**dimethylamino), among which **9d** exhibited excellent antitumor activity. This suggested that a bulkier substituent was favorable for the cytotoxic activity.

**Table 1:** *In vitro* cytotoxic activity of compounds **9a**–**f**, **13b**, **13d**, **13e** and **14a**–**f** *^a^* (IC_50_, µg/mL).

Compounds	NR_1_R_2_	ECA-109 *^b^*	A-549 *^c^*	HL-60 *^d^*	PC-3 *^e^*
**9a**	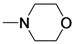	6.8	2.8	2.3	6.1
**9b**	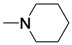	1.3	1.6	2.6	2.5
**9c**	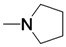	4.6	2.4	7.4	11.6
**9d**	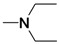	1.0	1.5	0.96	3.9
**9e**	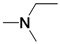	2.4	2.6	4.0	6.7
**9f**	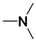	2.1	2.5	2.0	6.5
**13b**	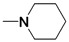	>50	>50	N.T.	N.T.
**13d**	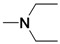	>50	>50	N.T.	N.T.
**13e**	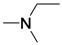	>50	34.1	N.T.	N.T.
**14a**	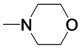	5.3	6.1	10.1	7.3
**14b**	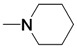	15.5	33.6	11.0	5.8
**14c**	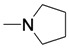	17.1	30.0	10.0	4.8
**14d**	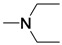	19.6	28.1	5.4	2.1
**14e**	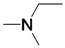	17.5	35.3	4.2	3.0
**14f**	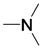	24.3	23.8	6.0	4.5
**Taxol**	-	12.8 × 10^−3^	13.6 × 10^−3^	2.2 × 10^−3^	5.3 × 10^−3^

*^a^* Data represent the mean values of three independent determinations; *^b^* Human esophageal carcinoma cell; *^c^* Human lung adenocarcinoma cell; *^d^* Human leukemia cell; *^e^* Human prostate carcinoma cell; N.T. not tested.

Because of the excellent activity of flavopiridol, the closed-ring derivatives **13** and **14** bearing the same substituents were synthesized and evaluated. Unfortunately, the formation of the B ring led to the loss of cytotoxic activity (compounds **13b**, **13d** and **13e**). Meanwhile, the importance of an exposed hydroxyl and the significant difference between dimethoxyflavone and hydroxyflavone cores (e.g., **13b** *vs.* **14b**, **13d** *vs.* **14d**, **13e** *vs.* **14e**) was found.

Hydroxyflavones **14a**–**f** displayed moderate cytotoxic activity, whereas dimethoxyflavones **13b**, **13d** and **13e** had only marginal or no cytotoxic effects against the tested cell lines. Another interesting observation is that the flavone derivatives **14a**–**f** with different substituent patterns at position-3'. displayed selective cytotoxic activity against PC-3, with IC_50_ values all lower than 10 µg/mL. Besides, the open chain aliphatic amino substituted flavones **14d**, **14e** and **14f** showed significant cytotoxic activity against the HL-60 cell line.

## 3. Experimental

### 3.1. Chemistry

Melting points are not corrected and were recorded on a Buchi apparatus. IR spectra (KBr pellets, 400–4000 cm^−1^) were recorded on a Bruker VECTOR 22 FTIR spectrophotometer. ^1^H-NMR spectra and NOE spectra were recorded on a Bruker AM 400 instrument at 400 MHz (chemical shifts are expressed as δ values relative to TMS as internal standard). Mass spectra (MS), ESI (positive) were recorded on an Esquire-LC-00075 spectrometer.

#### 3.1.1. 3-(2-Chlorophenyl)-1-(2-hydroxy-4,6-dimethoxyphenyl)prop-2-en-1-one (**4**)

To a stirred solution of **3** (0.39 g, 2 mmol) and 2-chlorobenzaldehyde (0.27 g, 3 mmol) in 80% EtOH (10 mL), KOH (1.0 g, 18 mmol) was added at 0 °C. The reaction mixture was stirred at room temperature overnight and then neutralized with 2N HCl and extracted with CH_2_Cl_2_ (2 × 30 mL). The combined organic fraction was washed with brine, dried over anhydrous Na_2_SO_4_, and concentrated under reduced pressure. The residue was recrystallized from EtOH to give 0.57 g (89%) of the title compound **4** as a yellow solid. m.p. 135–137 °C (Ref. [22]: m.p. 136–137 °C); IR (KBr): 3454 (OH), 2953, 2870 (CH_3_), 1631 (C=O), 1557 (C=C), 1274 (Ar-O), 1064 (C-O), 750 cm^−^^1^; ^1^H-NMR (CDCl_3_) δ: 14.20 (s, 1H, OH-2'), 8.14 (d, 1H, *J* = 15.6 Hz, H-β), 7.87 (d, 1H, *J* = 15.6 Hz, H-α), 7.69 (m, 1H, H-6), 7.43 (m, 1H, H-3), 7.30 (m, 2H, H-4 and 5), 6.11 (s, 1H, H-5'), 5.96 (s, 1H, H-3'), 3.90 (s, 3H, OCH_3_), 3.84 (s, 3H, OCH_3_). ESI-MS: *m/z* 319 [M+1]^+^.

#### 3.1.2. 1-(2-Allyloxy-4,6-dimethoxyphenyl)-3-(2-chlorophenyl)prop-2-en-1-one (**5**)

To a suspension of **4** (3.20 g, 10 mmol) and anhydrous K_2_CO_3_ in dry acetone, allyl bromide (9.1 mL, 10 mmol) was added with stirring. The resulting suspension was refluxed for 6 h, then the mixture was filtered, washed with acetone, and the filtrate was evaporated to dryness. The residue was purified by silica gel column chromatography using petroleum ether/EtOAc (6:1) as eluent to afford pure **5** (3.40 g, 95%) as a buff-colored liquid. IR (KBr): 3007 (=CH), 2944, 2849 (CH_3_, CH_2_), 1620 (C=O), 1535 (C=C), 1272 (Ar-O), 1081 (C-O) cm^−^^1^; ^1^H-NMR (CDCl_3_) δ: 7.79 (d, 1H, *J* = 16.0 Hz, H-β), 7.66 (m, 1H, H-6), 7.39 (m, 1H, H-3), 7.28 (m, 2H, H-4 and 5), 6.93 (d, 1H, *J* = 16.0 Hz, H-α), 6.16 (s, 1H, H-5'), 6.14 (s, 1H, H-3'), 5.95 (m, 1H, H-2"), 5.33 (dd, 1H, *J_1_* = 16.8 Hz, *J_2_* = 1.2 Hz, H-3"), 5.50 (m, 2H, H-1"), 3.84 (s, 3H, OCH_3_), 3.78 (s, 3H, OCH_3_). ESI-MS: *m/z* 359 [M+1]^+^.

#### 3.1.3. 1-(3-Allyl-2-hydroxy-4,6-dimethoxyphenyl)-3-(2-chlorophenyl)prop-2-en-1-one (**6**)

A solution of **5** (4.00 g, 11.2 mmol) in *N*,*N*-dimethylaniline (60 mL) under N_2_ was heated to reflux for 4 h. The solvent was removed *in vacuo*, and the residue was recrystallized from EtOH and gave 3.05 g (75%) of title compound **6** as a yellow solid. m.p. 167–168 °C; IR (KBr): 3448 (OH), 2979, 2945 (CH_3_, CH_2_), 1630 (C=O), 1581 (C=C), 1274 (Ar-O), 1135 (C-O) cm^−1^; ^1^H-NMR (CDCl_3_) δ: 13.93 (s, 1H, OH-2'), 8.06 (d, 1H, *J* = 15.6 Hz, H-β), 7.79 (d, 1H, *J* = 15.6 Hz, H-α), 7.65 (m, 1H, H-6), 7.39 (m, 1H, H-3), 7.26 (m, 2H, H-4 and 5), 5.97 (s, 1H, H-5'), 5.94 (m, 1H, H-2"), 4.95 (m, 2H, H-3"), 3.91 (s, 3H, OCH_3_), 3.87 (s, 3H, OCH_3_), 3.31 (m, 2H, H-1"). ESI-MS: *m/z* 359 [M+1]^+^.

#### 3.1.4. 2-Allyl-6-[3-(2-chlorophenyl)acryloyl]-3,5-dimethoxyphenyl acetate (**7**)

Acetic anhydride (1.27 mL, 13.5 mmol) was added to a solution of **6** (1.60 g, 5 mmol) in pyridine (20 mL). The reaction mixture was stirred at room temperature overnight, then neutralized with 2N HCl, and extracted with CH_2_Cl_2_ (3 × 40 mL). The combined organic extracts were washed with brine, dried over anhydrous Na_2_SO_4_, and concentrated under reduced pressure. The residue was purified by silica gel column chromatography using petroleum ether/EtOAc (5:1) as eluent to give pure **7** (1.70 g, 85%) as a yellow solid. m.p. 106–109 °C; IR (KBr): 2979, 2948, 2846 (CH_3_, CH_2_), 1762, 1660 (C=O), 1612 (C=C), 1205 (Ar-O), 1091 (C-O) cm^−^^1^; ^1^H-NMR (CDCl_3_) δ: 7.89 (d, 1H, *J* = 15.6 Hz, H-β), 7.65 (m, 1H, H-6), 7.40 (m, 1H, H-3), 7.30 (m, 2H, H-4 and 5), 7.06 (d, 1H, *J* = 15.6 Hz, H-α), 5.97 (s, 1H, H-5'), 5.90 (m, 1H, H-2"), 4.96 (m, 2H, H-3"), 3.91 (s, 3H, OCH_3_), 3.83 (s, 3H, OCH_3_), 3.32 (m, 2H, H-1"), 2.32 (s, 3H, COCH_3_). ESI-MS: *m/z* 401 [M+1]^+^.

#### 3.1.5. 2-[3-(2-Chlorophenyl)acryloyl]-3,5-dimethoxy-6-oxiranylmethylphenyl acetate (**8**)

To a stirred solution of **7** (0.60 g, 1.5 mmol) in CH_2_Cl_2_ (20 mL) was added a solution of *m*-chloroperoxybenzoic acid (0.42 g, 2.5 mmol) in CH_2_Cl_2_ (40 mL) dropwise during 0.5 h, with the temperature being maintained 0–5 °C. The mixture was stirred at room temperature for 24 h, and then the CH_2_Cl_2_ layer was washed successively with aqueous sodium bicarbonate and brine, dried over anhydrous Na_2_SO_4_, and concentrated under reduced pressure. The residue was purified by silica gel column chromatography using petroleum ether/EtOAc (6:1) as eluent to give pure **8** (0.42 g, 65%) as a yellow solid. m.p.106–109 °C; IR (KBr): 2943, 2842 (CH_3_, CH_2_), 1762, 1660 (C=O), 1612 (C=C), 1207 (Ar-O), 1090 (C-O), 768 cm^−1^; ^1^H-NMR (CDCl_3_) δ: 7.89 (d, 1H, *J* = 15.6 Hz, H-β), 7.65 (m, 1H, H-6), 7.41 (m, 1H, H-3), 7.33 (m, 2H, H-4 and 5), 7.01 (d, 1H, *J* = 15.6 Hz, H-α), 6.43 (s, 1H, H-5'), 3.92 (s, 3H, OCH_3_), 3.86 (s, 3H, OCH_3_), 3.07 (m, 1H, H-2"), 2.70 (dd, 1H, *J_1_* = 16.0 Hz, *J_2_* = 4.0 Hz, H-1"α), 2.67 (dd, 1H, *J_1_* = 15.6 Hz, *J_2_* = 4.4 Hz, H-1"β), 2.59 (t, 1H, *J* = 7.6 Hz, H-3"α), 2.51 (dd, 1H, *J_1_* = 5.6 Hz, *J_2_* = 2.4 Hz, H-3"β), 2.25 (s, 3H, COCH_3_). ESI-MS: *m/z* 417 [M+1]^+^.

#### 3.1.6. General Procedure to Obtain Target Chalcones **9a**–**f**

To a stirred solution of **8** (0.15 g, 0.36 mmol) and a suitable secondary amine (3.6 mmol) in CH_3_CN (2 mL), a catalytic amount of triethylamine (0.1 mL) was added. The resulting mixture was stirred at room temperature for 24 h. Then acetonitrile was removed *in vacuo*, extracted with CH_2_Cl_2_, the combined organic extracts were washed with brine, dried over anhydrous Na_2_SO_4_, and concentrated under reduced pressure. The residue was purified by silica gel column chromatography using petroleum ether/EtOAc/Et_3_N (100:100:2.5) as eluent to give pure target chalcones **9a**–**f**. In this manner, the following target chalcones were obtained.

*3-(2-Chlorophenyl)-1-{2-hydroxy-3-[2-hydroxy-3-(morpholino-4-yl)propyl]-4,6-dimethoxyphenyl}prop-2-en-1-one* (**9a**)*.* Yield: 72%, m.p. 141–143 °C; IR (KBr): 3458 (OH), 2920, 2851 (CH_3_, CH_2_), 1626 (C=O), 1566 (C=C), 1267 (Ar-O), 1172 (C-O), 758 cm^−^^1^; ^1^H-NMR (CDCl_3_) δ: 13.93 (s, 1H, OH-2'), 8.11 (d, 1H, *J* = 15.6 Hz, H-β), 7.81(d, 1H, *J* = 15.6 Hz, H-α), 7.69 (m, 1H, H-6), 7.43 (m, 1H, H-3), 7.30 (m, 2H, H-4 and 5), 6.01 (s, 1H, H-5'), 4.00 (s, 3H, OCH_3_), 3.95 (s, 3H, OCH_3_), 3.81 (m, 1H, H-2"), 3.70 (m, 4H, CH_2_O, CH_2_ × 2), 3.10 (dd, 1H, *J_1_* = 13.6Hz, *J_2_* = 7.2 Hz, H-1"α), 2.91 (dd, 1H, *J_1_* = 13.2 Hz, *J_2_* = 7.2 Hz, H-1"β), 2.49 (m, 2H, H-3"), 2.24 (m, 4H, NCH_2_, CH_2_ × 2). ESI-MS: *m/z* 462 [M+1]^+^.

*3-(2-Chlorophenyl)-1-{2-hydroxy-3-[2-hydroxy-3-(piperidin-1-yl)propyl]-4,6-dimethoxyphenyl} prop-2-en-1-one* (**9b**)*.* Yield: 75%, m.p. 135–136 °C; IR (KBr): 3443 (OH), 2936, 2887 (CH_3_, CH_2_), 1628 (C=O), 1611, 1576 (C=C), 1272 (Ar-O), 1107 (C-O), 754 cm^−^^1^; ^1^H-NMR (CDCl_3_) δ: 8.10 (d, 1H, *J* = 15.6 Hz, H-β), 7.81 (d, 1H, *J* = 15.6 Hz, H-α), 7.69 (m, H-6), 7.43 (m, 1H, H-3), 7.30 (m, 2H, H-4 and 5), 6.01 (s, 1H, H-5'), 3.94 (s, 3H, OCH_3_), 3.91 (s, 3H, OCH_3_), 3.91 (m, 1H, H-2"), 2.87 (dd, 1H, *J_1_* =13.6 Hz, *J_2_* = 6.4 Hz, H-1"α), 2.73 (dd, 1H, *J_1_* = 13.2 Hz, *J_2_* =6.8 Hz, H-1"β), 2.55 (m, 2H, H-3"), 2.32 (m, 4H, NCH_2_, CH_2_ × 2), 1.53 (m, 4H, NCH_2_CH_2_, CH_2_ × 2), 1.41 (m, 2H, CH_2_CH_2_CH_2_); ^13^C-NMR (DMSO-d_6_) δ: 192.2, 167.0, 164.1, 162.0, 141.1, 134.6, 133.3, 131.6, 130.8, 129.1, 128.4, 127.3, 108.9, 108.1, 90.2, 71.1, 60.7, 56.8, 56.6, 53.3, 33.2, 23.8, 21.2; ESI-MS: *m/z* 460 [M+1]^+^.

*3-(2-Chlorophenyl)-1-{2-hydroxy-3-[2-hydroxy-3-(pyrrolidin-1-yl)propyl]-4,6-dimethoxyphenyl}* *prop-2-en-1-one* (**9c**)*.* Yield: 55%, m.p. 132–135 °C; IR (KBr): 3453 (OH), 2937, 2879 (CH_3_, CH_2_), 1628 (C=O), 1601, 1576 (C=C), 1274 (Ar-O), 1110 (C-O), 754 cm^−^^1^; ^1^H-NMR (CDCl_3_) δ: 13.95 (s, 1H, OH-2'), 8.10 (d, 1H, *J* = 15.6 Hz, H-β), 7.79 (d, 1H, *J* = 15.6 Hz, H-α), 7.69 (m, 1H, H-6), 7.43 (m, 1H, H-3), 7.31 (m, 2H, H-4 and 5), 6.02 (s, 1H, H-5'), 3.94 (s, 3H, OCH_3_), 3.91 (s, 3H, OCH_3_), 3.91 (m, 1H, H-2"), 2.87 (dd, 1H, *J_1_* =13.2 Hz, *J_2_* = 5.6 Hz, H-1"α), 2.79 (dd, 1H, *J_1_* = 13.2 Hz, *J_2_* =5.8 Hz, H-1"β), 2.69 (m, 4H, NCH_2_, CH_2_ × 2), 2.52 (m, 2H, H-3"), 1.73 (s, 4H, NCH_2_CH_2_, CH_2_ × 2). ESI-MS: *m/z* 446 [M+1]^+^.

*3-(2-Chlorophenyl)-1-[3-(3-diethylamino-2-hydroxypropyl)-2-hydroxy-4,6-dimethoxyphenyl]prop-2-en-1-one* (**9d**)*.* Yield: 63%, m.p. 114–116 °C; IR (KBr): 3450 (OH), 2936, 2859 (CH_3_, CH_2_), 1625 (C=O), 1579 (C=C), 1272 (Ar-O), 1123 (C-O), 759 cm^−^^1^; ^1^H-NMR (CDCl_3_) δ: 13.94 (s, 1H, OH-2'), 8.10 (d, 1H, *J* = 15.6 Hz, H-β), 7.84 (d, 1H, *J* = 15.6 Hz, H-α), 7.74 (m, 1H, H-6), 7.43 (m, 1H, H-3), 7.31 (m, 2H, H-4 and 5), 5.98 (s, 1H, H-5'), 3.97 (s, 3H, OCH_3_), 3.90 (s, 3H, OCH_3_), 3.92 (m, 1H, H-2"), 3.03 (dd, 1H, *J_1_* =13.6 Hz, *J_2_* =7.2 Hz, H-1"α), 2.84 (dd, 1H, *J_1_* = 13.6 Hz, *J_2_* = 6.8 Hz, H-1"β), 2.39 (m, 6H, H-3" and NCH_2_), 0.94 (t, 6H, NCH_2_CH_3_, CH_3_ × 2); ^13^C-NMR (DMSO-d_6_) δ: 192.5, 169.6, 164.9, 162.1, 140.9, 134.2, 132.3, 130.3, 129.8, 127.2, 127.1, 126.8, 108.0, 105.1, 90.5, 70.8, 61.0, 55.2, 54.6, 47.7, 34.2, 12.2; ESI-MS: *m/z* 448 [M+1]^+^.

*3-(2-Chlorophenyl)-1-[3-(3-ethylmethylamino-2-hydroxypropyl)-2-hydroxy-4,6-dimethoxyphenyl]* *prop-2-en-1-one* (**9e**)*.* Yield: 76%, m.p. 115–118 °C; IR (KBr): 3448 (OH), 2924, 2856 (CH_3_, CH_2_), 1628 (C=O), 1563 (C=C), 1271 (Ar-O), 1120 (C-O), 749 cm^−^^1^; ^1^H-NMR (CDCl_3_) δ: 8.12 (d, 1H, *J* = 15.6 Hz, H-β), 7.82 (d, 1H, *J* = 15.6 Hz, H-α), 7.70 (m, 1H, H-6), 7.44 (m, 1H, H-3), 7.30 (m, 2H, H-4 and 5), 6.02 (s, 1H, H-5'), 3.95 (s, 3H, OCH_3_), 3.92 (s, 3H, OCH_3_), 3.92 (m, 1H, H-2"), 2.88 (dd, 1H, *J_1_* =13.2Hz, *J_2_* = 5.2 Hz, H-1"α), 2.74 (dd, 1H, *J_1_* = 13.6 Hz, *J_2_* = 4.8 Hz, H-1"β), 2.41 (m, 4H, H-3" and NCH_2_), 2.24 (s, 3H, NCH_3_), 1.04 (t, 3H, NCH_2_CH_3_). ESI-MS: *m/z* 434 [M+1]^+^.

*3-(2-Chlorophenyl)-1-[3-(3-dimethylamino-2-hydroxypropyl)-2-hydroxy-4,6-dimethoxyphenyl] prop-2-en-1-one* (**9f**)*.* Yield: 75%, m.p. 127–128 °C; IR (KBr): 3458 (OH), 2914, 2866 (CH_3_, CH_2_), 1629 (C=O), 1611, 1566 (C=C), 1256 (Ar-O), 1137 (C-O), 769 cm^−^^1^; ^1^H-NMR (CDCl_3_) δ: 14.03 (s, 1H, OH-2'), 8.12 (d, 1H, *J* = 15.6 Hz, H-α), 7.82 (d, 1H, *J* = 15.6 Hz, H-β), 7.69 (m, 1H, H-6), 7.43 (m, 1H, H-3), 7.30 (m, 2H, H-4 and 5), 6.02 (s, 1H, H-5'), 3.95 (s, 3H, OCH_3_), 3.92 (s, 3H, OCH_3_), 3.92 (m, 1H, H-2"), 2.87 (dd, 1H, *J_1_* = 13.6Hz, *J_2_* = 7.2 Hz, H-1"α), 2.75 (dd, 1H, *J_1_* = 13.6 Hz, *J_2_* = 6.8 Hz, H-1"β), 2.44 (t, 1H, *J* = 12.6 Hz, H-3"α), 2.26 (s, 6H, NCH_3_, CH_3_ × 2), 2.21 (dd, 1H, *J_1_* = 13.6 Hz, *J_2_* = 6.8 Hz, H-3"β). ESI-MS: *m/z* 420 [M+1]^+^.

#### 3.1.7. 8-Allyl-2-(2-chlorophenyl)-5,7-dimethoxy-2,3-dihydro-4*H*-1-benzopyran-4-one (**10**)

To a solution of **6** (4.52 g, 12.6 mmol) in 95% EtOH (136 mL) was added NaOAc (12.90, 157 mmol). The mixture was heated at reflux for 24 h, then EtOH was removed *in vacuo*, H_2_O was added and the mixture was extracted with CH_2_Cl_2_ (3 × 50 mL). The combined organic extracts were washed with brine, dried over anhydrous Na_2_SO_4_, and concentrated under reduced pressure. The residue was purified by silica gel column chromatography using petroleum ether/EtOAc (5:1) as eluent to afford pure flavanone **10** (3.84 g, 85%) as a colorless solid, m.p. 131–133 °C. IR (KBr): 3006 (C=CH), 2942, 2879 (CH_3_, CH_2_), 1676 (C=O), 1602, 1567 (C=C), 1265 (Ar-O), 1126 (C-O) cm^−^^1^; ^1^H-NMR (CDCl_3_) δ: 7.70 (m, 1H, H-6'), 7.26 (m, 3H, H-3', 4' and 5'), 6.15 (s, 1H, H-6), 5.90 (m, 1H, H-2"), 5.73 (dd, 1H, *J_1_* = 13.2 Hz, *J_2_* = 3.2 Hz, H-2), 4.95 (m, 2H, H-2"), 3.96 (s, 3H, OCH_3_), 3.91 (s, 3H, OCH_3_), 3.35 (m, 2H, H-1"), 2.99 (dd, 1H, *J_1_* = 16.8 Hz, *J_2_* = 3.6 Hz, H-3'α), 2.75 (dd, 1H, *J_1_* = 16.4 Hz, *J_2_* = 4.0 Hz, H-3'β). ESI-MS: *m/z* 359 [M+1]^+^.

#### 3.1.8. 8-Allyl-2-(2-chlorophenyl)-5,7-dimethoxy-4*H*-1-benzopyran-4-one (**11**)

To a stirred solution of **10** (2.1 g, 5.85 mmol) in pyridine (42 mL) was added I_2_ (1.49 g, 5.86 mmol). The mixture was heated to 90 °C for 6 h, and then pyridine was removed *in vacuo*, followed by addition of solid Na_2_SO_3_ until fading of the color. The mixture was extracted with CH_2_Cl_2_ (3 × 50 mL); the combined organic extracts were washed with brine, dried over anhydrous Na_2_SO_4_, and concentrated under reduced pressure. The residue was purified by silica gel column chromatography using petroleum ether/EtOAc (2:1) as eluent to give pure flavone **11** (1.00 g, 49%) as a colorless solid, m.p. 181–183 °C. IR (KBr): 3015 (C=CH), 2943, 2879 (CH_3_, CH_2_), 1663 (C=O), 1602, 1573 (C=C), 1217 (Ar-O), 1063 (C-O) cm^−^^1^; ^1^H-NMR (CDCl_3_) δ: 7.63 (m, 1H, H-6'), 7.52 (m, 1H, H-3'), 7.39 (m, 2H, H-4' and 5'), 6.54 (s, 1H, H-3), 6.46 (s, 1H, H-6), 5.92 (m, 1H, H-2"), 4.95 (m, 2H, H-3"), 4.02 (s, 3H, OCH_3_), 3.97 (s, 3H, OCH_3_), 3.54 (m, 2H, H-1"). ESI-MS: *m/z* 357 [M+1]^+^.

#### 3.1.9. 2-(2-Chlorophenyl)-5,7-dimethoxy-8-oxiranylmethyl-4*H*-1-benzopyran-4-one (**12**)

To a stirred solution of **11** (3.28 g, 10 mmol) in CH_2_Cl_2_ (40 mL) was added a solution of *m*-chloroperoxybenzoic acid (0.42 g, 2.5 mmol) in CH_2_Cl_2_ (80 mL) dropwise during 0.5 h, and the mixture was stirred at room temperature for 24 h, then the CH_2_Cl_2_ layer was washed successively with aqueous sodium bicarbonate and brine, dried over anhydrous Na_2_SO_4_, and concentrated under reduced pressure. The residue was purified by silica gel column chromatography using petroleum ether/EtOAc (2:1) as eluent to give pure **12** (1.78 g, 68%) as colorless solid. m.p. 106–109 °C. IR (KBr): 2999, 2886 (CH_3_, CH_2_), 1645 (C=O), 1603, 1579 (C=C), 1265 (Ar-O), 1116 (C-O) cm^−^^1^; ^1^H-NMR (CDCl_3_) δ: 7.70 (m, 1H, H-6'), 7.28 (m, 3H, H-3', 4' and 5'), 6.52 (s, 1H, H-3), 6.47 (s, 1H, H-6), 4.01 (s, 3H, OCH_3_), 3.96 (s, 3H, OCH_3_), 3.30 (dd, 1H, *J_1_* = 13.6 Hz, *J_2_* = 6.0 Hz, H-1"α), 3.16 (m, 1H, H-2"), 2.90 (dd, 1H, *J_1_* = 13.6 Hz, *J_2_* = 6.4 Hz, H-1"β), 2.69 (t, 1H, *J* = 5.0 Hz, H-3"α), 2.51 (m, 1H, *J_1_* = 4.8 Hz, *J_2_* = 2.0 Hz, H-3"β). ESI-MS: *m/z* 373 [M+1]^+^.

#### 3.1.10. General Procedure to Obtain Dimethoxyflavones **13a**–**f**

To a stirred solution of **12** (0.19 g, 0.50 mmol) and a suitable secondary amine (5.0 mmol) in ethanol (2 mL), a catalytic amount of triethylamine (0.1 mL) was added. The resulting mixture was refluxed for 24 h. Then ethanol was removed *in vacuo*, and the residue was then extracted with CH_2_Cl_2_. The CH_2_Cl_2_ extracts were washed with brine, dried over anhydrous Na_2_SO_4_, and concentrated under reduced pressure. The residue was purified by silica gel column chromatography using EtOAc/EtOH/Et_3_N (100:2:2.5) as eluent to afford pure target chalcones **13a**–**f**. In this manner, the following target dimethoxyflavones were obtained:

*2-(2-Chlorophenyl)-8-[2-hydroxy-3-(morpholino-4-yl)propy]-5,7-dimethoxy-4H-1-benzopyran-4-one* (**13a**)*.* Yield: 75%, m.p. 145–147 °C; IR (KBr): 3427 (OH), 2939, 2874 (CH_3_, CH_2_), 1645 (C=O), 1612, 1601 (C=C), 1285 (Ar-O), 1066 (C-O) cm^−^^1^; ^1^H-NMR (CDCl_3_) δ: 7.68 (m, 1H, H-6'), 7.54 (m, 1H, H-3'), 7.39 (m, 2H, H-4' and 5'), 6.52 (s, 1H, H-3), 6.49 (s, 1H, H-6), 5.00 (brs, 1H, OH-2''), 4.04 (s, 3H, OCH_3_), 3.98 (s, 3H, OCH_3_), 4.01 (m, 1H, H-2"), 3.69 (m, 4H, CH_2_O, CH_2_ × 2), 3.10 (dd, 1H, *J_1_* =13.6 Hz, *J_2_* =7.2 Hz, H-1"α), 2.91 (dd, 1H, *J_1_* = 13.2 Hz, *J_2_* = 7.2 Hz, H-1"β), 2.49 (m, 2H, H-3"), 2.24 (m, 4H, NCH_2_, CH_2_ × 2). ESI-MS: *m/z* 460 [M+1]^+^.

*2-(2-Chlorophenyl)-8-[2-hydroxy-3-(piperidin-1-yl)propyl]-5,7-dimethoxy-4H-1-benzopyran-4-one* (**13b**)*.* Yield: 78%, m.p. 149–153 °C; IR (KBr): 3434 (OH), 2932, 2847 (CH_3_, CH_2_), 1655 (C=O), 1612, 1600 (C=C), 1284 (Ar-O), 1120 (C-O), 770 cm^−^^1^; ^1^H-NMR (CDCl_3_) δ: 7.66 (m, 1H, H-6'), 7.52 (m, 1H, H-3'), 7.39 (m, 2H, H-4' and 5'), 6.52 (s, 1H, H-3), 6.46 (s, 1H, H-6), 4.00 (s, 3H, OCH_3_), 3.96 (s, 3H, OCH_3_), 3.95 (m, 1H,H-2"), 3.07 (dd, 1H, *J_1_* =13.6 Hz, *J_2_* = 6.4 Hz, H-1"α), 2.86 (dd, 1H, *J_1_* = 13.2 Hz, *J_2_* =6.8 Hz, H-1"β), 2.50 (brs, 1H, OH-2''), 2.32 (m, 2H, H-3"), 2.25 (m, 4H, NCH_2_, CH_2_ × 2), 1.52 (m, 4H, NCH_2_CH_2_, CH_2_ × 2), 1.39 (m, 2H, CH_2_CH_2_CH_2_). ESI-MS: *m/z* 458 [M+1]^+^.

*2-(2-Chlorophenyl)-8-[2-hydroxy-3-(pyrrolidin-1-yl)propyl]-5,7-dimethoxy-4H-1-benzopyran-4-one* (**13c**)*.* Yield: 74%, m.p. 149–153 °C; IR (KBr): 3428 (OH), 2963, 2933 (CH_3_, CH_2_), 1643 (C=O), 1602 (C=C), 1376 (Ar-O), 1127 (C-O) cm^−^^1^; ^1^H-NMR (CDCl_3_) δ: 7.67 (m, 1H, H-6'), 7.52(m, 1H, H-3'), 7.40 (m, 2H, H-4' and'), 6.52 (s, 1H, H-3), 6.46 (s, 1H, H-6), 4.04 (s, 3H, OCH_3_), 4.00 (s, 3H, OCH_3_), 4.02 (m, 1H, H-2"), 3.06 (dd, 1H, *J_1_* =13.6 Hz, *J_2_* = 6.8 Hz, H-1"α), 2.92 (dd, 1H, *J_1_* = 13.6 Hz, *J_2_* =6.8 Hz, H-1"β), 2.31 (m, 6H, NCH_2_ × 2 and H-3"), 1.73 (s, 4H, NCH_2_CH_2_, CH_2_ × 2). ESI-MS: *m/z* 444 [M+1]^+^.

*2-(2-Chlorophenyl)-8-(3-diethylamino-2-hydroxypropyl)-5,7-dimethoxy-4H-1-benzopyran-4-one* (**13d**). Yield: 80%, m.p. 149–153 °C; IR (KBr): 3435 (OH), 2964, 2931 (CH_3_, CH_2_), 1654 (C=O), 1621, 1602 (C=C), 1384 (Ar-O), 1118 (C-O), 772 cm^−^^1^; ^1^H-NMR (CDCl_3_) δ: 7.69 (m, 1H, H-6'), 7.52 (m, 1H, H-3'), 7.36 (m, 2H, H-4' and 5'), 6.53 (s, 1H, H-3), 6.42 (s, 1H, H-6), 4.01 (s, 3H, OCH_3_), 3.97 (s, 3H, OCH_3_), 3.90 (m, 1H, H-2"), 3.07 (dd, 1H, *J_1_* = 13.2 Hz, *J_2_* = 6.4 Hz, H-1"α), 2.84 (dd, 1H, *J_1_* = 13.2 Hz, *J_2_* = 6.4 Hz, H-1"β), 2.39 (m, 6H, H-3" and NCH_2_), 0.94 (t, 6H, NCH_2_CH_3_, CH_3_ × 2); ^13^C-NMR (DMSO-d_6_) δ: 179.3, 165.3, 164.2, 161.9, 157.0, 134.2, 132.9, 130.1, 129.8, 128.0, 126.5, 125.2, 112.5, 107.1, 95.8, 70.8, 61.0, 56.2, 55.9, 47.5, 35.8, 12.6; ESI-MS: *m/z* 446 [M+1]^+^.

*2-(2-Chlorophenyl)-8-(3-ethylmethylamino-2-hydroxypropyl)-5,7-dimethoxy-4H-1-benzopyran-4-one* (**13e**)*.* Yield: 82%, m.p. 149–153 °C; IR (KBr): 3424 (OH), 2968, 2938 (CH_3_, CH_2_), 1653 (C=O), 1602, 1578 (C=C), 1328 (Ar-O), 1120 (C-O), 763 cm^−^^1^; ^1^H-NMR (CDCl_3_) δ: 7.67 (m, 1H, H-6'), 7.51 (m, 1H, H-3'), 7.36 (m, 2H, H-4' and 5'), 6.52 (s, 1H, H-3), 6.45 (s, 1H, H-6), 4.01 (s, 3H, OCH_3_), 3.97 (s, 3H, OCH_3_), 3.92 (m, 1H, H-2"), 3.07 (dd, 1H, *J_1_* = 13.6 Hz, *J_2_* = 6.8 Hz, H-1"α), 2.85 (dd, 1H, *J_1_* = 13.2 Hz, *J_2_* = 6.8 Hz, H-1"β), 2.41 (m, 4H, H-3" and NCH_2_), 2.17 (s, 3H, NCH_3_), 0.96 (t, 3H, NCH_2_CH_3_). ESI-MS: *m/z* 432 [M+1]^+^.

*2-(2-Chlorophenyl)-8-(3-dimethylamino-2-hydroxypropyl)-5,7-dimethoxy-4H-1-benzopyran-4-one* (**13f**)*.* Yield: 87%, m.p. 149–153 °C; IR (KBr): 3441 (OH), 2939 (CH_3_, CH_2_), 1653 (C=O), 1623, 1602 (C=C), 1327 (Ar-O), 1120 (C-O), 762 cm^−^^1^; ^1^H-NMR (CDCl_3_) δ: 7.66 (m, 1H, H-6'), 7.51 (m, 1H, H-3'), 7.38 (m, 2H, H-4' and 5'), 6.51 (s, 1H, H-3), 6.46 (s, 1H, H-6), 4.00 (s, 3H, OCH_3_), 3.97 (s, 3H, OCH_3_), 3.92 (m, 1H, H-2"), 3.05 (dd, 1H, *J_1_* = 13.6 Hz, *J_2_* = 6.8 Hz, H-1"α), 2.86 (dd, 1H, *J_1_* = 13.2 Hz, *J_2_* = 3.8 Hz, H-1"β), 2.37 (t, 1H, *J* =11.8 Hz, H-3"α), 2.18 (s, 6H, NCH_3_, CH_3_ × 2), 2.11 (dd, 1H, *J_1_* = 12.4 Hz, *J_2_* = 3.2 Hz, H-3"β). ESI-MS: *m/z* 418 [M+1]^+^.

#### 3.1.11. General Procedure to Obtain Monomethoxyflavones **14a**–**f**

To a stirred solution of dimethoxyflavone (0.2 mmol) in 1,2-dichloroethane (2 mL) at room temperature was added a solution of BBr_3_ (1 mol/L, 0.4 mL, 0.4 mmol). The resulting mixture was stirred at room temperature for 3 h. It was cooled to 0 °C, and quenched by water (10 mL). It was made alkaline with Na_2_CO_3_ solution and extracted with CH_2_Cl_2_. The CH_2_Cl_2_ extracts were washed with brine, dried over anhydrous Na_2_SO_4_, and concentrated under reduced pressure. The residue was purified by silica gel column chromatography using EtOAc/petroleum ether/Et_3_N (100:50:2.5) as eluent to give pure monomethoxyflavones **14a**–**f**. In this manner, the following targets were obtained:

*2-(2-Chlorophenyl)-5-hydroxy-8-[2-hydroxy-3-(morpholin-4-yl)propyl]-7-methoxy-4H-1-benzopyran-4-one* (**14a**)*.* Yield: 88%, m.p. 139–151 °C; IR (KBr): 3424 (OH), 2934, 2870 (CH_3_, CH_2_), 1658 (C=O), 1620, 1601 (C=C), 1278 (Ar-O), 1166 (C-O) cm^−^^1^; ^1^H-NMR (CDCl_3_) δ: 12.80 (s, 1H, OH-5), 7.66 (m, 1H, H-6'), 7.54 (m, 1H, H-3'), 7.42 (m, 2H, H-4' and 5'), 6.54 (s, 1H, H-6), 6.45 (s, 1H, H-3), 3.98 (m, 1H, H-2"), 3.92 (s, 3H, OCH_3_), 3.64 (m, 4H, -CH_2_-O, CH_2_ × 2), 3.03 (dd, 1H, *J_1_* = 13.6 Hz, *J_2_* = 6.8 Hz, H-1"α), 2.85 (dd, 1H, *J_1_* = 13.6 Hz, *J_2_* = 5.6 Hz, H-1"β), 2.56 (m, 2H, H-3"), 2.30 (m, 4H, -N-CH_2_, CH_2_ × 2). ESI-MS: *m/z* 446 [M+1]^+^.

*2-(2-Chlorophenyl)-5-hydroxy-8-[2-hydroxy-3-(piperidin-1-yl)propyl]-7-methoxy-4H-1-benzopyran-4-one* (**14b**)*.* Yield: 85%, m.p. 127–128 °C; IR (KBr): 3430 (OH), 2930, 2845 (CH_3_, CH_2_), 1659 (C=O), 1612, 1600 (C=C), 1280 (Ar-O), 1121(C-O), 771 cm^−^^1^; ^1^H-NMR (CDCl_3_) δ: 12.80 (s, 1H, OH-5), 7.68 (m, 1H, H-6'), 7.51(m, 1H, H-3'), 7.42 (m, 2H, H-4' and 5'), 6.53 (s, 1H, H-3), 6.42 (s, 1H, H-6), 3.89 (m, 4H, OCH_3_ and H-2"), 3.56 (brs, 1H, OH-2"), 3.00 (dd, 1H, *J_1_* =13.6 Hz, *J_2_* = 6.0 Hz, H-1"α), 2.79 (dd, 1H, *J_1_* = 13.2 Hz, *J_2_* =6.0 Hz, H-1"β), 2.47 (m, 2H, H-3"), 2.23 (m, 4H, NCH_2_, CH_2_ × 2), 1.48 (m, 4H, NCH_2_CH_2_, CH_2_ × 2), 1.37 (m, 2H, CH_2_CH_2_CH_2_). ESI-MS: *m/z* 444 [M+1]^+^.

*2-(2-Chlorophenyl)-5-hydroxy-8-[2-hydroxy-3-(pyrrolidin-1-yl)propyl]-7-methoxy-4H-1-benzopyran-4-one* (**14c**)*.* Yield: 72%, m.p. 134–137 °C; IR (KBr): 3424 (OH), 2963, 2933 (CH_3_, CH_2_), 1659 (C=O), 1602 (C=C), 1384 (Ar-O), 1117 (C-O), 765 cm^−^^1^; ^1^H-NMR (CDCl_3_) δ: 12.79 (s, 1H, OH-5), 7.67 (m, 1H, H-6'), 7.51 (m, 1H, H-3'), 7.37 (m, 2H, H-4' and 5'), 6.53 (s, 1H, H-3), 6.42 (s, 1H, H-6), 3.88 (s, 3H, OCH_3_), 3.78 (m, 1H, H-2"), 3.54 (brs, 1H, OH-2"), 3.00 (dd, 1H, *J_1_* = 13.6 Hz, *J_2_* = 4.8 Hz, H-1"α), 2.78 (dd, 1H, *J_1_* = 13.6 Hz, *J_2_* = 4.8 Hz, H-1"β), 2.48 (m, 2H, H-3"), 2.22 (s, 4H, NCH_2_, CH_2_ × 2), 1.73 (s, 4H, NCH_2_CH_2_, CH_2_ × 2). ESI-MS: *m/z* 430 [M+1]^+^.

*2-(2-Chlorophenyl)-8-(3-diethylamino-2-hydroxypropyl)-5-hydroxy-7-methoxy-4H-1-benzopyran-4-one* (**14d**)*.* Yield: 77%, m.p. 114–115 °C; IR (KBr): 3437 (OH), 2962, 2929 (CH_3_, CH_2_), 1662 (C=O), 1619, 1600 (C=C), 1294 (Ar-O), 1114 (C-O), 775 cm^−^^1^; ^1^H-NMR (CDCl_3_) δ: 12.79 (s, 1H, OH-5), 7.69 (m, 1H, H-6'), 7.53 (m, 1H, H-3'), 7.40 (m, 2H, H-4' and 5'), 6.53 (s, 1H, H-3), 6.42 (s, 1H, H-6), 4.01 (m, 1H, H-2"), 3.90 (s, 3H, OCH_3_), 3.06 (dd, 1H, *J_1_* =13.4 Hz, *J_2_* = 6.4 Hz, H-1"α), 2.84 (dd, 1H, *J_1_* = 13.2 Hz, *J_2_* =6.4 Hz, H-1"β), 2.39 (m, 6H, H-3" and NCH_2_), 0.94 (t, 6H, NCH_2_CH_3_, CH_3_ × 2); ^13^C-NMR (DMSO-d_6_) δ: 183.3, 166.3, 165.2, 162.9, 157.0, 134.2, 132.9, 131.1, 130.8, 129.0, 126.9, 125.2, 108.5, 106.1, 95.8, 70.4, 60.8, 56.2, 55.9, 47.6, 36.2, 12.4; ESI-MS: *m/z* 432 [M+1]^+^.

*2-(2-Chlorophenyl)-8-(3-ethylmethylamino-2-hydroxypropyl)-5-hydroxy-7-methoxy**-**4H-1-benzopyran-4-one* (**14e**)*.* Yield: 70%, m.p. 119–123 °C; IR (KBr): 3425 (OH), 2965, 2945 (CH_3_, CH_2_), 1662 (C=O), 1617, 1583 (C=C), 1332 (Ar-O), 1114 (C-O) cm^−^^1^; ^1^H-NMR (CDCl_3_) δ: 12.80 (s, 1H, OH-5), 7.70 (m, 1H, H-6'), 7.54 (m, 1H, H-3'), 7.44 (m, 2H, H-4' and 5'), 6.56 (s, 1H, H-3), 6.45 (s, 1H, H-6), 3.92 (s, 3H, OCH_3_), 3.90 (m, 1H, H-2"), 3.02 (dd, 1H, *J_1_* = 12.6 Hz, *J_2_* = 6.4 Hz, H-1"α), 2.82 (dd, 1H, *J_1_* = 13.0 Hz, *J_2_* = 6.4 Hz, H-1"β), 2.42 (m, 4H, H-3" and NCH_2_), 2.17 (s, 3H, NCH_3_), 0.98 (t, 3H, NCH_2_CH_3_). ESI-MS: *m/z* 418 [M+1]^+^.

*2-(2-Chlorophenyl)-8-(3-dimethylamino-2-hydroxypropyl)-5-hydroxy-7-methoxy-4H-1-benzopyran-4-one* (**14f**)*.* Yield: 80%, m.p. 149–153 °C; IR (KBr): 3435 (OH), 2937 (CH_3_, CH_2_), 1662 (C=O), 1617, 1583 (C=C), 1287 (Ar-O), 1120 (C-O) cm^−^^1^; ^1^H-NMR (CDCl_3_) δ: 12.82 (s, 1H, OH-5), 7.69 (m, 1H, H-6'), 7.54 (m, 1H, H-3'), 7.45 (m, 2H, H-4' and 5'), 6.55 (s, 1H, H-3), 6.45 (s, 1H, H-6), 3.92 (s, 3H, OCH_3_), 3.91 (m, 1H, H-2"), 3.02 (dd, 1H, *J_1_* = 13.6 Hz, *J_2_* = 3.8 Hz, H-1"α), 2.83 (dd, 1H, *J_1_* = 13.2 Hz, *J_2_* = 3.2 Hz, H-1"β), 2.36 (t, 1H, *J* = 12.0 Hz, H-3"α), 2.20 (s, 6H, NCH_3_, CH_3_ × 2), 2.12 (dd, 1H, *J_1_* = 12.0 Hz, *J_2_* = 3.2 Hz, H-3"β); ^13^C-NMR (DMSO-d_6_) δ: 183.5, 166.0, 165.0, 162.8, 157.1, 134.6, 133.2, 131.4, 130.8, 128.4, 126.9, 124.8, 107.5, 105.7, 95.6, 70.2, 63.8, 56.0, 44.6, 36.0; ESI-MS: *m/z* 404 [M+1]^+^. 

### 3.2. Cytotoxicity Assays

*In vitro* cytotoxicity assays were carried out using 96-well microplate cultures and MTT staining based on the reported method [23]. Briefly, the cells were cultured in RPMI-1640 medium supplemented with fetal bovine serum in 5% CO_2_ incubated at 37 °C. For the experiment, the drugs were dissolved in DMSO. After seeding 4,000 cells per well in a 96-well microplate, the cells were treated with the sample at four concentrations per compound. The controls received just DMSO. Cells were then incubated at 37 °C for 3 days, after which time 5 μL MTT in citrate buffer was added. Cells were stored again for 4 h. After removing the medium and adding DMSO (100 μL per well) into the microplate with shaking for 10 min, the formazan crystals (the product of MTT reacting with dehydrogenase existing in the mitochondria) were redissolved and the absorbance was measured on a model MR 7000 microtiter plate reader (Dynatech International Corporation, Edgewood, NY, USA) at a wavelength of 570 nm. The result was expressed as the drug concentration which inhibited cell growth by 50% as compared to the control (IC_50_). The IC_50_ values were calculated from regression lines obtained from the probit of the percent cell growth inhibition plotted as a function of the logarithm of the dose. The tumor cell lines panel consisted of ECA-109, A-549, HL-60 and PC-3. In all of these experiments, three replicate wells were used to determine each point and taxol was used as the positive control.

## 4. Conclusions

We have designed and synthesized a novel series of flavonoid derivatives bearing diverse aliphatic amino moieties. Their antitumor activities were evaluated against the ECA-109, A-549, HL-60, and PC-3 cancer cell lines using the MTT method and a preliminary SAR was described. We identified two chlacone derivatives **9b** and **9d** as the most promising candidates which have high potency with IC_50_ values lower than 5 µg/mL against all human tumor cell lines examined. Further investigations will focus on the introduction of other chalcone templates and investigation of their effects on cytotoxic activities.

## References

[1] Middleton E., Kandaswami C., Theoharides T.C. (2000). The effects of plant flavonoids on mammalian cells: Implications for inflammation, heart disease, and cancer. Pharmacol. Rev..

[2] Ren W.Y., Qiao Z.H., Wang H.W., Zhu L., Zhang L. (2003). Flavonoids: Promising anticancer agents. Med. Chem. Res..

[3] Li Y., Fang H., Xu W. (2007). Recent advance in the research of flavonoids as anticancer agents. Mini Rev. Med. Chem..

[4] Min K., Ebeler S.E. (2009). Quercetin inhibits hydrogen peroxide-induced DNA damage and enhances DNA repair in Caco-2 cells. Food Chem. Toxicol..

[5] Lee B.W., Lee J.H., Lee S.T., Lee W.S., Jeong T.S., Park K.H. (2005). Antioxidant and cytotoxic activities of xanthones from Cudraruatricuspidata. Bioorg. Med. Chem. Lett..

[6] Lopez-Lazaro M., Willmore E., Austin C.A. (2010). The dietary flavonoids myricetin and fisetin act as dual inhibitors of DNA topoisomerases I and II in cells. Mutation. Res-Gen. Tox. Ens..

[7] Kelland L.R. (2000). Flavopiridol, the first cyclin-dependent kinase inhibitor to enter the clinic: Current status. Expert Opin. Investig. Drugs.

[8] Senderowicz A.M., Sausville E.A. (2000). Preclinical and clinical development of cyclin-dependent kinase modulators. J. Natl. Cancer Inst..

[9] Murthi K.K., Dubay M., McClure C., Brizuela L., Boisclair M.D., Worland P.J., Mansuri M.M., Pal K. (2000). Structure-activity relationship studies of flavopiridol analogues. Bioorg. Med. Chem. Lett..

[10] Dauzonne D., Folleas B., Martinez L., Chabot G.G. (1997). Synthesis and *in vitro* cytotoxicity of a series of 3-aminoflavones. Eur. J. Med. Chem..

[11] Xia Y., Yang Z.Y., Xia P., Bastow K.F., Nakanishi Y., Lee K.H. (2000). Antitumor agents. Part 202: Novel 2'-amino chalcones: Design, synthesis and biological evaluation. Bioorg. Med. Chem. Lett..

[12] Nielsen S.F., Larsen M., Boesen T., Schonning K., Kromann H. (2005). Cationic chalcone antibiotics. Design, Synthesis, And mechanism of action. J. Med. Chem..

[13] Liu X.L., Go M.L. (2006). Antiproliferative properties of piperidinylchalcones. Bioorg. Med. Chem..

[14] Chen Z.W., Hu Y.Z., Wu H.H., Jiang H.D. (2004). Design and synthesis of 3-(2-pyridyl)pyrazolo[1,5-*a*]pyrimidines as potent CRF1 receptor antagonists. Bioorg. Med. Chem. Lett..

[15] Ichino K., Tanaka H., Ito K., Tanaka T., Mizuno M. (1988). Synthesis of helilandin B, pashanone, and their isomers. J. Nat. Prod..

[16] Sekizaki H. (1988). Synthesis of 2-Benzylidene-3(2H)-benzofuran-3-ones (Aurones) by oxidation of 2’-hydroxychalcones with mercury(II) acetate. Bull. Chem. Soc. Jpn..

[17] Parmar V.S., Gupta S., Sharma R.K., Sharma V.K. (1990). Synthesis of 2,3-dihydro-8-(3-hydroxy-3-methylbut-1-enyl)-7-methoxy-2-phenyl-4H-1-benzopyran-4-one: A novel structure for falciformin. J. Org. Chem..

[18] Maitrejean M., Comte G., Barron D., Kirat K.E., Conseil G., Pietro A.D. (2000). The flavanolignan silybin and its hemisynthetic derivatives, a novel series of potential modulators of *p*-glycoprotein. Bioorg. Med. Chem. Lett..

[19] Chu H.W., Wu H.T., Lee Y.J. (2004). Regioselective hydroxylation of 2-hydroxychalcones by dimethyldioxirane towards polymethoxylated flavonoids. Tetrahedron.

[20] Dao T.T., Chi Y.S., Kim J., Kim H.P., Kim S., Park H. (2003). Synthesis and PGE_2_ inhibitory activity of 5,7-dihydroxyflavones and their *o*-methylated flavone analogs. Arch. Pharm. Res..

[21] Tabaka A.C., Murthi K.K., Pal K., Teleha C.A. (1999). Application of modified flavone closure for the preparation of racemic L86–8275. Org. Process Res. Dev..

[22] Boeck P. (2005). Antifungal activity and studies on mode of action of novel xanthoxyline-derived chalcones. Arch. Pharm..

[23] Cardenas M., Marder M., Blank V.C., Roguin L.P. (2006). Antitumor activity of some natural flavonoids and synthetic derivatives on various human and murine cancer cell lines. Bioorg. Med. Chem..

